# Use of a basophil activation test as a complementary diagnostic tool in the diagnosis of severe peanut allergy in adults

**DOI:** 10.1186/s13601-015-0064-9

**Published:** 2015-06-11

**Authors:** Georgios Rentzos, Vanja Lundberg, Christina Lundqvist, Rui Rodrigues, Jenny van Odijk, Anna-Carin Lundell, Teet Pullerits, Esbjörn Telemo

**Affiliations:** Sahlgrenska University Hospital, Section of Allergology, Gothenburg, Sweden; Department of Rheumatology and Inflammation Research, Sahlgrenska Academy, University of Gothenburg, Gothenburg, Sweden; Department of Clinical Immunology and Transfusion Medicine, Sahlgrenska University Hospital, Gothenburg, Sweden; Department of Respiratory Medicine and Allergology, Section of Allergology, Sahlgrenska University Hospital, 413 45 Gothenburg, Sweden

**Keywords:** Peanut allergy, Basophil activation test, Allergen components

## Abstract

**Background:**

Diagnosis of severe peanut allergy is difficult and delays in making an accurate diagnosis may place the patient at risk. Adults with a history of anaphylaxis must strictly avoid any contact with peanuts or products that may contain traces of peanuts. For these persons, conventional evaluations with skin prick testing (SPT) and IgE tests may not be sufficient to assess the risk of anaphylaxis. Therefore, we investigated whether the basophil activation test (BAT) could be used for the diagnosis of severe peanut allergy in adults. We compared the non-invasive BAT with conventional laboratory diagnostic tests, including SPT and specific IgE to allergen extracts and components, for the diagnosis of severe peanut allergy.

**Methods:**

Forty-seven persons with severe allergy to peanuts and a clinical diagnosis of anaphylaxis (PA-group), 22 subjects with peanut sensitization (PS-group) and 22 control (C-group) subjects, all in the age range of 18–60 years, were recruited retrospectively and prospectively into the study. Thirty-four patients with peanut allergy and 11 peanut-sensitized patients were sensitized to soy, while 36 patients in the PA-group and 20 patients in the PS-group were sensitized to birch pollen. All the patients and control subjects were investigated with BAT and SPT for responses to peanut, soy and birch extracts and their serum samples were assayed for the presence of specific IgE to peanut, soy and birch extracts, as well as IgE to allergen components (ISAC).

**Results:**

In a multivariate factor analysis, severe peanut allergy (PA) was positively associated with SPT to peanut, IgE to peanut, BAT to peanut and IgE to rAra h 1, 2, 3 and 6 peanut components, as well as to soy components (nGly m 5 and nGly m 6). In contrast, peanut sensitization was positively associated with increased levels of IgE to rAra h 8, birch and birch-related components. BAT-detected reactivity to peanut was significantly higher in patients who had a history of severe allergy to peanuts, as compared with patients who were sensitized to peanuts (*p* < 0.001), and the receiver operating curve (ROC) analysis showed that BAT had high sensitivity and specificity for predicting severe peanut allergy, with a ROC area under the curve of 0.862. However, in the PA-group, the BAT results for peanut correlated only weakly with the levels of IgE to rAra h 1, 2 and 3 and nAra h 6. Study limitations: oral provocation in the patients with a history of severe peanut allergy could not be performed to compare clinical reactivity with the BAT result due to ethical constraints. Neither was it possible to perform BAT with peanut recombinant allergens which were not available at the time the study commenced

**Conclusions:**

BAT is useful in determining the severity of peanut allergy and may be used as a complementary diagnostic tool to ensure accurate diagnosis of severe peanut allergy in adults. Thus, it may reduce the need to subject these patients to further tests, including an open challenge with peanuts.

**Electronic supplementary material:**

The online version of this article (doi:10.1186/s13601-015-0064-9) contains supplementary material, which is available to authorized users.

## Background

Peanut allergy is one of the most common food allergies among children and shows increasing prevalence in adults [1–5]. The conventional tests for diagnosing peanut allergy are not adequate to predict the severity of the allergy, which means that they are not optimal for the correct diagnosis of asymptomatic patients who are sensitized to peanuts [3, 4]. Accurate clinical assessment requires further investigation with an oral food challenge, which may not be desirable for safety reasons [6]. The lack of accurate diagnostic tools may lead to incorrect or misleading diagnoses, with consequent impaired quality of life for the patient [7]. Although most food allergies become less severe or resolve entirely during adulthood, the skin prick and/or IgE-tests continue to give positive results for some of these patients. In some regions, such as Central and Northern Europe, the diagnostic issues related to peanut allergy are even more complex due to the cross-reaction with birch pollen [8]. A person who is allergic to birch pollen may develop a secondary peanut allergy, which may be interpreted as a true primary peanut allergy [9]. Moreover, individuals who are sensitized to peanuts may develop secondary allergies to other legumes, *e.g.*, soy bean and *vice versa* [10–12].

Some years ago, a complementary diagnostic tool (microarray technique) was introduced that determines the sensitization profile with the help of allergen components [13, 14], and this has been used mainly to resolve sensitization patterns in food allergy. Through the use of the peanut allergen components Ara h 1, 2, 3, 6, 8 and 9, the differential diagnosis of true clinical peanut allergy has improved [15, 16]. However, while the pattern of sensitization to the different peanut allergens may predict that the patient is going to react to peanuts, it cannot predict the severity of the reaction [17]. The profile of sensitization to recombinant allergens provides additional help in understanding the cross-reactivity patterns of different allergens *e.g.* legumes, albeit without ascertaining whether this cross-reactivity has any clinical relevance.

A relatively new and promising diagnostic tool, the basophil activation test (BAT) [18], has been applied recently for the diagnosis of various allergies; initially, BAT was used mainly for the diagnosis of food allergies in children [19–23]. The BAT has also gained attention because it seems to show a good correlation with true or persistent peanut allergy in children [24]. Whether the reactivity measured by BAT correlates with asymptomatic sensitization or persistent clinical allergy in adults remains to be elucidated. Similarly, only limited information is available on whether the BAT correlates with the outcome of an oral food challenge [25]. Interestingly, the BAT was used in a recent study to monitor tolerance induction in children who underwent oral immunotherapy with egg [26], and basophil histamine release has been used to monitor the effectiveness of anti-IgE (omalizumab) treatment administered prior to an oral immunotherapy regime in patients with severe peanut allergy [27].

The aim of the presents study was to investigate whether patients who have suffered a severe allergic reaction to peanuts or who have been designated as being allergic to peanuts since childhood, can be diagnosed with clinical or persistent peanut allergy using the BAT. We also examined whether the BAT could discriminate between patients with severe peanut allergy and sensitized patients with no or mild symptoms in order to evaluate if a person is no longer severely allergic to peanut even when displaying persistent IgE-mediated peanut sensitivity, as assessed using conventional tests, including reactivities to allergen components. In addition, we asked if the BAT can be used for the diagnosis of co-existent concomitant allergy to soy in patients who are sensitized or allergic to peanuts and if any possible underlying clinical cross-reactivity among legumes can be revealed by combining BAT reactivity and IgE sensitization profiling to allergen components.

## Materials and methods

### Study population

Forty-seven adults with severe allergy to peanuts (PA-group), 22 peanut-sensitized persons (PS-group) and 22 healthy controls (C-group), all in the age range of 18–60 years, were recruited either retrospectively or prospectively to the study between January and December of 2013. All the patients had been referred to the Allergy Clinic at the Sahlgrenska University Hospital in Gothenburg for allergy investigation. The PA-group consisted of patients with severe peanut allergy who had a convincing history of anaphylaxis to peanuts with objective symptoms, together with the routine allergological investigation including a detailed clinical history and/or IgE titers to rAra h 2 > 0.35 kU/L. The PS-group consisted of patients who had previously undergone investigations with oral peanut challenge for suspected peanut allergy due to a co-existing allergy to birch pollen. The majority of the patients in the PA-group and PS-group were also sensitized to soy and/or birch pollen. The profiles of the participants, including the clinical diagnosis, grade of reaction to peanut and peanut avoidance, are presented in Table 1. The non-allergic control subjects were recruited among the staff at the Sahlgrenska University Hospital or their relatives and friends. There were no drop-outs during the study. All patients and controls were investigated using the SPT for peanut, soy and birch, as well as measurements of total IgE and specific IgE for peanut, soy and birch. Blood was collected from each subject for the BAT and serum was saved for analysis of sensitization to allergen components (ISAC). Exclusion criteria for all the subjects were: pregnancy; lactation; rheumatic or systemic disease; and immune deficiency. Five patients in the PA-group and three in the PS-group had previous or currently ongoing treatment with immunotherapy for pollen allergy (birch and grass allergy). All the patients in the PS-group answered a questionnaire regarding whether they had eaten peanuts after they had undergone a negative open oral challenge with peanut.

**Table 1:** Clinical and demographic features of the patient groups included in the study (PA=peanut allergic patients, PS=peanut sensitized patients, C=healthy controls)

	PA (*n* = 47)	PS (*n* = 22)	C (*n* = 22)
Men	25	7	5
Women	22	15	11
Age (mean)	25	28	33
Sensitization to peanut (sIgE)	47	20	-
Sensitization to peanut (SPT)	47	18	-
Sensitization to birch (sIgE)	36	20	-
Sensitization to birch (SPT)	34	18	-
Sensitization to soy (IgE)	34	11	-
Sensitization to soy (SPT)	19	13	-
Asthma	31	11	-
Immunotherapy (birch pollen)	5	3	-
Open peanut challenge during allergy investigation	8	22	-
Peanut avoidance	47	7	-
Soy avoidance	19	5	-

All the allergic patients were taking intermittent medications, such as anti-histamines, nasally administered corticosteroids and eye-drops during the pollen season. Patients with asthma used inhaled corticosteroids and beta-mimetics as required. None of the patients required medication with oral or injected corticosteroids during the period of the study.

This study was approved by the Ethics Committee of the Regional Ethical Review Board in Gothenburg (Dnr. 591-10). Written informed consent was obtained from both the patients and control subjects.

### Allergy assessments

#### Skin prick test (SPT)

The skin prick test was performed using allergen extracts from peanut, soy and birch (Soluprick, ALK-Abelló, Hørsholm, Denmark). Histamine (10 mg/ml) and vehicle were used as references. The SPT was considered positive when the wheal reaction diameter was ≥3 mm. A wheal reaction equivalent in diameter to that of the histamine reference was recorded as 3+, while one that was half of the diameter of the histamine reaction was recorded as 2+. If the wheal diameter was twice that of the histamine reference it was recorded as 4+, whereas one that was four times that of the reference was recorded as 5+ *etc.*

#### ImmunoCAP

The serum levels of total IgE and specific IgE antibodies to peanut (f13), soy (f14), and birch-pollen allergen extracts (t3) were measured using ImmunoCAP (Thermofisher Scientific, Uppsala, Sweden).

#### ISAC

IgE against allergen components was measured using a micro-array Immunoassay/ImmunoCap (ISAC; Thermofisher Scientific, Uppsala, Sweden), which covers 112 components from 51 sources of allergens. The results are expressed as ISAC Standardized Units (ISU) with a threshold of >0.3 ISU. The ISAC analysis was performed as recommended by the manufacturer.

#### Open challenge

All the patients in the PS-group underwent an open challenge with peanut and showed a negative outcome before they were included in the study. The open challenges were performed as part of the investigation according to EAACI position paper and the total dose of peanut used for the open challenge was 10 g [5].

### Allergen extracts used for the basophil activation test

The allergen extracts used in this study were: peanut (*Arachis hypogaea*, protein concentration 6 mg/ml); soy (*Glycine max*, protein concentration of 1.9 mg/ml); and birch (*Betula verrucosa*, protein concentration of0.08 mg/ml) (Soluprick, ALK-Abelló, Hørsholm, Denmark). For the BAT, the allergen extracts were serially diluted in 10-fold steps from an initial 1/30 dilution of the Soluprick extract. The peanut extract was tested in 12 serial 10-fold dilutions and the soy and birch extracts were tested in five serial 10-fold dilutions. For the serum samples from some patients, the BAT was repeated with additional dilutions.

### Basophil activation test

Basophil activation was measured based on the up-regulation of CD63 on CD203c + basophils observed in flow cytometry of blood samples collected in heparinized tubes. All the tests were carried out within 4 h of blood sampling. To study the activation of basophils, the BasoFlowEx® Kit (EXBIO, Prague, Czech Republic) was used according to the manufacturer’s recommendations. Briefly, 100 μl of heparinized whole blood and 50 μl of Stimulation Buffer were added to all the tubes. Subsequently, 5 μl of allergen solution (allergen extracts for peanut, soy and birch; Soluprick) were added to the samples. For the positive control, 10 μl of Stimulation Control [a cross-linking anti-IgE antibody mixed with a stimulating peptide, N-formyl-Met-Leu-Phe (fMLP)] was added. The tubes were gently vortexed and incubated at 37 °C for 15 min in a water bath, followed by mixing with 20 μl of Staining Reagent, which contained anti-CD63 FITC and anti-CD203c PE antibodies. After further incubation for 20 min on ice, 300 μl of Lysing Solution were added and the tubes were re-incubated for 5 min at room temperature, followed by the addition of 4 ml of de-ionized water for 10 min. After centrifugation at 300 × *g* for 5 min, the supernatant fluid was removed and the pelleted cells were re-suspended in 0.4 ml PBS. Samples were analyzed in the BD FACSCanto II flow cytometer. The gate for the basophil population (CD203c ^positive^, SSC ^low^) was set as shown in Fig. 3. Using the negative control sample, the gate for non-stimulated basophils (CD63 ^low^) was set as shown in Fig. 3d. Basophils in Q2 were considered to be activated. The cut-off for determining a positive test was set at 15 % CD63-positive basophils, in line with the manufacturer’s instructions. The gates were the same for all the tests conducted on an individual patient, although they were positioned individually for each patient. The level of basophil activation is expressed as %CD63^+^ basophils above the threshold set in the negative control. Approximately 600 basophils were acquired for each analysis (Fig. 3d and e).

### Statistical analyses

The statistical analyses were carried out using the IBM SPSS Statistics 22.0 software. The values shown represent individual data-points or means and median values. For each patient, the basophil allergen threshold sensitivity was calculated as the lowest allergen concentration that was able to activate 50 % of the basophils that were activated in the stimulation control (BAT AC50). The BAT AC50-value was calculated using a linear interpolation of the response to the allergen and is presented as the log_10_ value of the dilution factor, *i.e.*, the higher the number the more sensitive is the patient. Data for BAT are reported as medians with interquartile ranges (IQR). Mann-Whitney U-tests were used for statistical comparisons of the groups of patients. Correlations between different parameters within the same group were evaluated by Spearman’s correlation coefficient. The complete results of the most relevant and influential statistical correlations between the variables in PA-group and the PS-group obtained in the study are provided in Additional file 1: Table S3 and Additional file 2: Table S4.

**Fig. 1:**
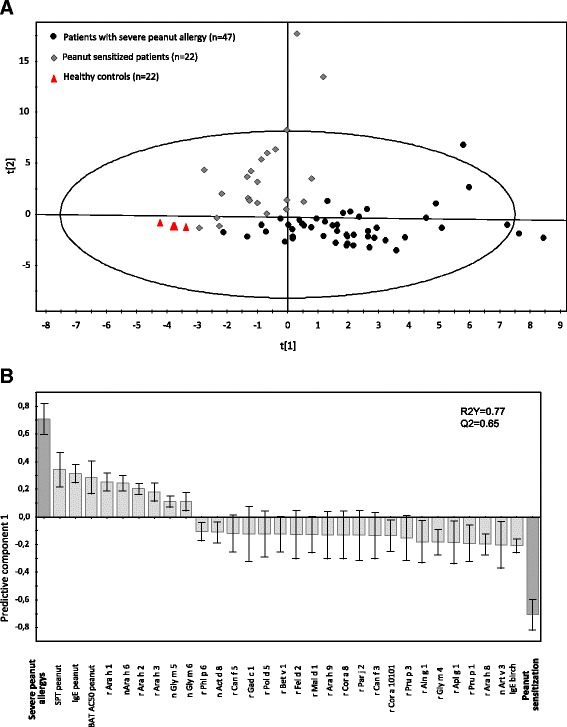
**a** PLS-discriminant analysis score scatter plot showing the distinction of patients with severe allergy to peanuts (black dots, n = 47) from patients sensitized to peanuts (green diamonds, n = 22). All the healthy controls are clustered in the lower-left quadrant (red triangles, n = 22). **b** OPLS-discriminant analysis column loadings plot depicting the associations between patients with severe allergy to peanuts and patients sensitized to peanuts. The X-variables represented by a bar pointing in the same direction as severe allergy to peanuts (located to the far left) are positively associated. The OPLS-DA column plot is based on X-variables with VIP-values ≥0.83. R2Y indicates how well the variation of Y is explained, while Q2 indicates how well Y can be predicted

**Fig. 2:**
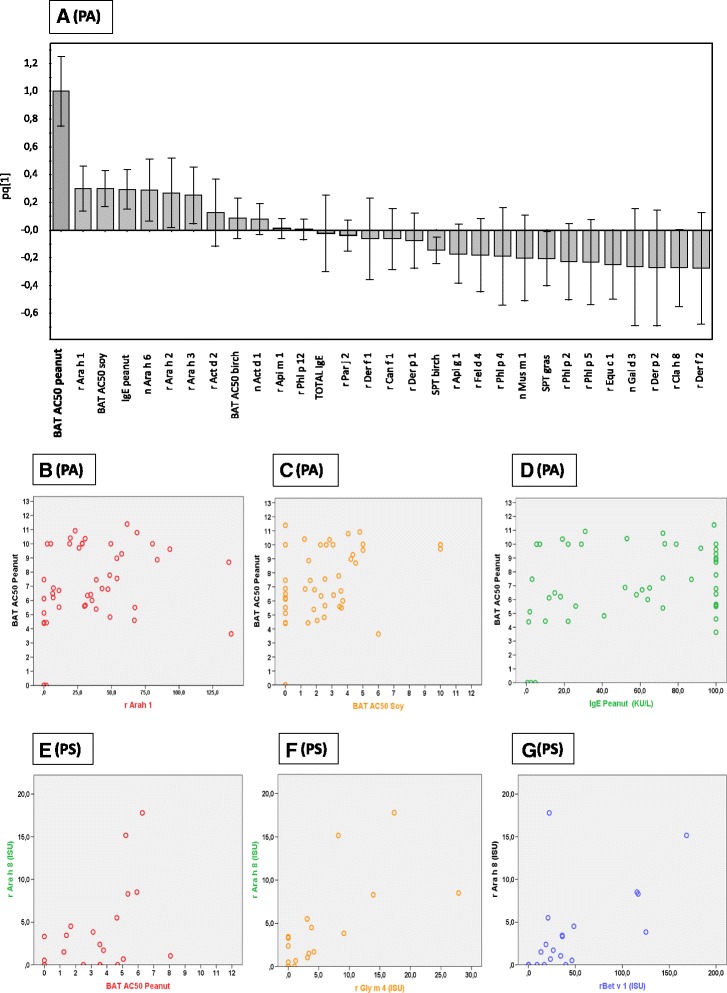
**a** OPLS column-loading plot showing the X-variables that are most strongly associated with the BAT AC50-value for peanut within the PA-group. X-variables represented by a bar pointing in the same direction as AC50 peanut (located to the far left) are positively associated, whereas variables in the opposite direction are inversely related. The OPLS plot is based on X-variables with VIP-values ≥0.88. R2Y indicates how well the variation of Y is explained, while Q2 indicates how well Y can be predicted. The univariate correlations between the variables most strongly associated with BAT AC50P in the PA-group are illustrated for: rAra h 1 (*r* = 0.314, *p* = 0.032) (**b**); BAT AC50 soy (*r* = 0.413, *p* = 0.04) (**c**); and IgE to peanut (*r* = 0.235, *p* = 0.112) (**d**). In the PS-group, the univariate correlations between rAra h 8 are illustrated for: BAT AC50P (*r* = 0.479, *p* = 0.024) (**e**); rGly m 4 (*r* = 0.757, *p* < 0.001) (**f**); and rBet v 1 (*r* = 0.676, *p* = 0.001) (**g**). PA, patients with severe peanut allergy; PS, peanut-sensitized patients

Logistic regression analysis was performed to determine whether covariate diagnostic variables could be combined with the BAT results to achieve a more accurate diagnosis. All tests were two-tailed and the level of significance was set at *p* < 0.05. Receiver operating characteristics (ROC) curve analysis was performed to calculate the optimal cutoff value of AC50 that corresponded to the highest specificity and sensitivity. Multivariate factor analysis (SIMCA-P+ software; Umetrics, Umeå, Sweden) was used to examine the relationships between individuals with severe peanut allergy or subjects sensitized to peanut (Y-variables) and the various parameters studied (X-variables). Projection to latent structures discriminant analysis (PLS-DA) was implemented to examine whether allergic individuals compared with sensitized and control individuals could be discriminated based on the X-variables examined. Orthogonal partial least-squares discriminant analysis (OPLS-DA) was performed to correlate Y-variables and X-variables to each other in linear multivariate models. Variable influence on projection (VIP) values can be used to discriminate between important and unimportant predictors for the model. The OPLS-DA plot of the results (Fig. 1b) is based on X-variables with variable influences on projection values (VIP-values) ≥0.83 and the OPLS column loading plot in Fig. 2a is based on VIP-values ≥0.88. In the OPLS analyses, the relative importance of each X-variable to the Y-variable is represented by column bars. The larger the bar and smaller the error bar and the stronger and more certain is the contribution to the model. The most influential X-variables were used for subsequent statistical analyses.

## Results

### Patients’ characteristics

The demographic and clinical characteristics of the patients and control subjects are shown in Table 1. Three patients in the PA-group and one patient in the PS-group who showed spontaneous stimulation of >10 % of their basophils in the negative control were excluded from the study. One patient in the PA-group was found to be a non-responder in the positive control of the BAT and was excluded from the study. Three healthy controls (C-group) showed a BAT response of less than 15 % of the level in the positive control but were still included in the study. In contrast, three patients in the PA-group and five patients in the PS-group were non-responders to peanut with less than 15 % of their basophils responding. Furthermore, 7/22 (32 %) peanut-sensitized subjects who had shown a negative open challenge test still avoided peanuts and 19/47 (42 %) patients with severe allergy to peanut avoided soy (Table 1).

### Peanut allergy can be distinguished from peanut sensitization in a multiple regression model that includes conventional tests and BAT to peanut

Initially, we investigated whether patients with severe allergy to peanuts (PA-group), patients sensitized to peanuts (PS-group) and healthy control subjects (C-group) could be discriminated based on X-variables that included the SPT (to peanut, soy and birch), total and specific (ISAC) IgE levels and basophil activation. PLS-DA demonstrated good distinction between the PA-group and PS-group (Fig. 1a). The majority of the PA patients appeared in the lower-right quadrant, while the PS patients were plotted in the upper-left quadrant. All the healthy controls were tightly clustered in the lower-left quadrant (Fig. 1a). Five individuals were located outside the ellipse, due to variability or missing data for one or more X-variables, compared to the other observations.

The X-variables that displayed the strongest relationship (positive or negative) to the PA and PS subjects, respectively, were identified in the OPLS-DA column plot (Fig. 1b). The model is based on X-variables with VIP-values ≥0.83. The VIP column plot for all X-variables assessed is shown in Additional file 3: Figure S1. X-variables represented by a bar pointing in the same direction as PA are positively associated, whereas X-variables pointing in the opposite direction are related to PS. Severe allergy to peanuts was positively associated with SPT to peanut, specific IgE to peanut, BAT AC50 to peanut and Ara h components 1, 2, 3 and 6 (Fig. 1b). Positively associated with being sensitized to peanut were specific IgE to birch and rAra h 8, along with other birch homolog variables (PR10-proteins) (Fig. 1b). Thus, these results indicate that patients with allergy to peanuts and patients sensitized to peanuts differ with respect to the magnitudes of their responses in conventional laboratory tests, as well as in the BAT to peanut. Therefore, the BAT in combination with all relevant variables might be useful for identifying cases of severe peanut allergy.

Among all X-variables examined, the variables most strongly positively associated with the BAT AC50 for peanut were IgE specific for peanut and the rAra h 1, 2, 3 and nAra h 6 components, along with the BAT AC50 for soy (Fig. 2a).

**Fig. 3:**
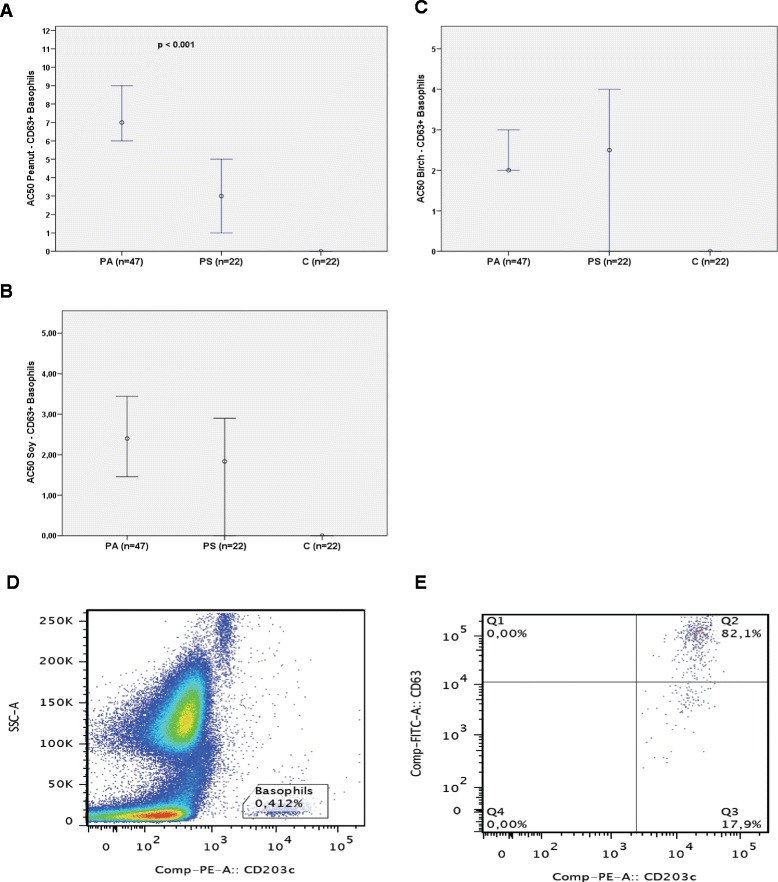
The median BAT AC50-value (50 % of maximal basophil stimulation) for (**a**) peanut, (**b**) soy and (**c**) birch allergen extract in the basophil activation test. PA, patients with severe peanut allergy; PS, peanut-sensitized patients; C, healthy controls. **d-e** Gating strategy for flow cytometry. **d** Basophils are gated as SSC ^low^ and CD203c ^positive^. **e** Using the negative control sample, a gate for non-stimulated basophils is set as CD63 ^low^. Cells in Q3 are considered to be non-activated basophils and cells in Q2 are considered to be activated basophils

### Basophil activation test results for peanut-allergic versus peanut-sensitized patients

The median BAT AC50 value obtained for peanut was significantly higher for the PA-group at 6.84 (IQR 4.50) than for the PS-group at 3.55 (IQR 4.15) (*p* < 0.001) (Fig. 3a). In the PA-group, there were 3 (6 %) non-responders to peanut and 5 (23 %) in the PS-group. No significant differences were noted comparing the median BAT values for birch or soy between the two groups (Fig. 3b and c, respectively). Basophils from healthy controls did not respond to any of the allergens tested (Fig. 3a–c). The gating strategy for the flow cytometry is presented in Fig. 3d and e.

### Does peanut BAT outcome correlate with other allergy parameters in peanut-allergic patients?

In the PA-group, there was a positive correlation between the BAT AC50 to peanut and BAT AC50 to soy (*r* = 0.413, *p* = 0.004) but no correlation to the BAT AC50 for birch (*r* = 0.161, *p* = 0.280). Interestingly, in this group, we found a rather weak correlation between the BAT AC50 for peanut and the levels of IgE directed against the individual peanut components rAra h 1 (*r* = 0.314, *p* = 0.032), rAra h 2 (*r* = 0.291, *p* = 0.047), rAra h 3 (*r* = 0.289, *p* = 0.049), and nAra h 6 (*r* = 0.347, *p* = 0.017), and surprisingly, no correlation with the SPT or IgE level to peanut nor with total serum IgE.

### Basophil activation test to soy and birch in peanut allergic vs peanut sensitized patients

In the PA-group, there were positive correlations between the BAT AC50 for soy and specific IgE to soy (*r* = 0.585, *p* < 0.001), as well as specific IgE directed against peanut (*r* = 0.584, *p* < 0.01). There was a similar correlation to the individual component nGly m 6 (*r* = 0.583, *p* < 0.01) but a weaker correlation to nGly m 5 (*r* = 0.391, *p* < 0.01), as well as to the peanut components rAra h 1 (*r* = 0.508 *p* < 0.001), rAra h 2 (*r* = 0.476, *p* < 0.001), rAra h 3 (*r* = 0.661, *p* < 0.001), and nAra h 6 (*r* = 0.484, *p* = 0.001).

In the same group, we observed positive correlations between the BAT AC50 for birch and r Bet v1 (*r* = 0.685, *p* < 0.01), SPT birch (*r* = 0.517, *p* < 0.01), IgE birch (*r* = 0.657, *p* < 0.01), and rAra h 8 (*r* = 0.417, *p* < 0.01).

In the PS-group, there were positive correlations between the BAT AC50 for peanut and the BAT AC50 for soy (*r* = 0.689, *p* < 0.01), but also the BAT AC50 for birch (*r* = 0.735, *p* < 0.01). In this group, the BAT reactivity to peanut correlated only with IgE to rAra h 8 (*r* = 0.479, *p* = 0.024) and rGly m 4 (*r* = 0.638, *p* = 0.01). Interestingly, in this group, a correlation was also found between the BAT AC50 for soy and the level of IgE to peanut (*r* = 0.470, *p* = 0.027), as well as to the soy component rGly m 4 (*r* = 0.447, *p* = 0.037).

A complete list of the significant statistical correlations observed between the studied variables in both groups is provided in Additional file 1: Table S3 and Additional file 2: Table S4 for the PA-group and PS-group, respectively.

### Can BAT discriminate between peanut-allergic and peanut-sensitized patients?

**Fig. 4:**
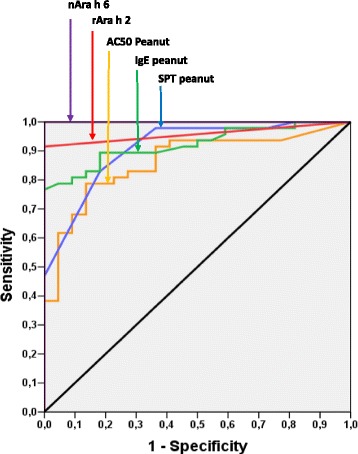
ROC curve assessments of the sensitivity and specificity of the basophil activation test for peanut (orange) (AUC = 0.862), SPT peanut (blue) (AUC = 0.910), IgE peanut (green) (AUC = 0.922), rAra h 2 (red) (AUC = 0.957), and nAra h 6 (purple) (AUC = 1.0)

To determine a cut-off level for reactivity to peanut in the BAT, so as to distinguish between the patients in the PA-group and PS-group, ROC curves were applied. They revealed that an optimal sensitivity of 79 % and a specificity of 86 % could be obtained at a BAT AC50 of 5.27, which would allow the diagnosis of patients with severe peanut allergy (AUC 0.862). With SPT to peanuts, the highest sensitivity of 83 % and highest specificity of 82 % were obtained for a wheal diameter of 3.5 (AUC 0.910), and the IgE to peanuts showed a sensitivity of 81 % and specificity of 91 % at an IgE level of 11.5 kU/L (AUC 0.922). The parameters that showed the greatest power for distinguishing the two groups in the ISAC assay were IgE to rAra h 2 [sensitivity of 91.5 % and specificity of 100 % at an IgE level of 0.75 ISU (AUC 0.957)], and nAra h 6 [sensitivity 100 % and specificity 100 % at a level of 1.16 ISU(AUC 1.0)] (Fig. 4).

### SPT and IgE reactivity

**Fig. 5:**
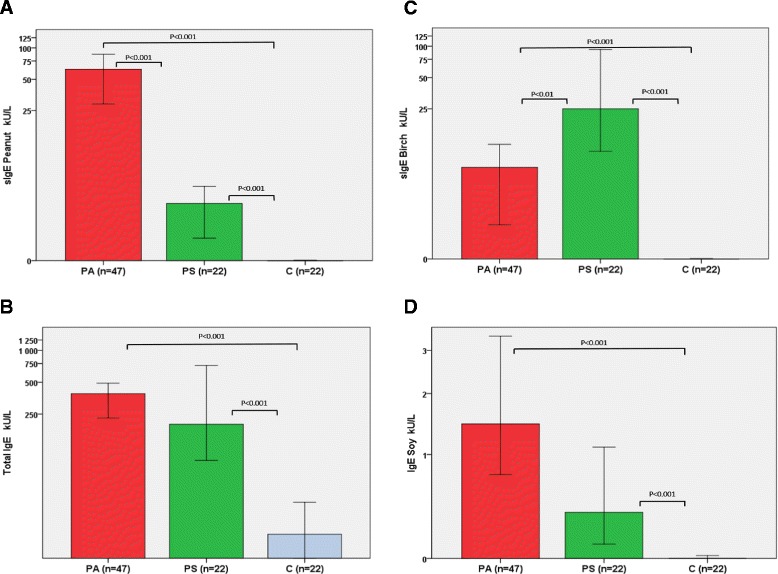
The median concentrations (kU/L) of specific IgE for (**a**) peanut, (**b**) soy and (**c**) birch and (**d**) the total IgE in patients with severe peanut allergy compared to sensitized patients and controls. PA, patients with severe peanut allergy; PS, peanut-sensitized patients; C, healthy controls. The Mann-Whitney test was used for the statistical comparisons

The SPT results, the median level of specific IgE and total IgE for the different groups are presented in Additional file 4: Table S2 and Additional file 5: Table S1. The median wheal diameter in the SPT for peanut was 4+ and the median level of specific IgE for peanut was 64 kU/L (IQR 82) in the PA-group, which was significantly higher than the SPT for peanut at 2+ (p < 0.001) and median level of specific IgE at 2.45 kU/L (IQR 3.4) in the PS-group (p < 0.001) (Additional file 5: Table S1 and Additional file 4: Table S2 and Fig. 5a). No significant differences were noted for total IgE and IgE for soy when comparing the PA-group and PS-group (Fig. 5b and d). However, the median level of specific IgE for birch was significantly higher (*p* = 0.01) in the PS-group at 25 kU/L (IQR 84.2) than in the PA-group at 6.1 kU/L (IQR 17.5) (Fig. 5c).

**Fig. 6:**
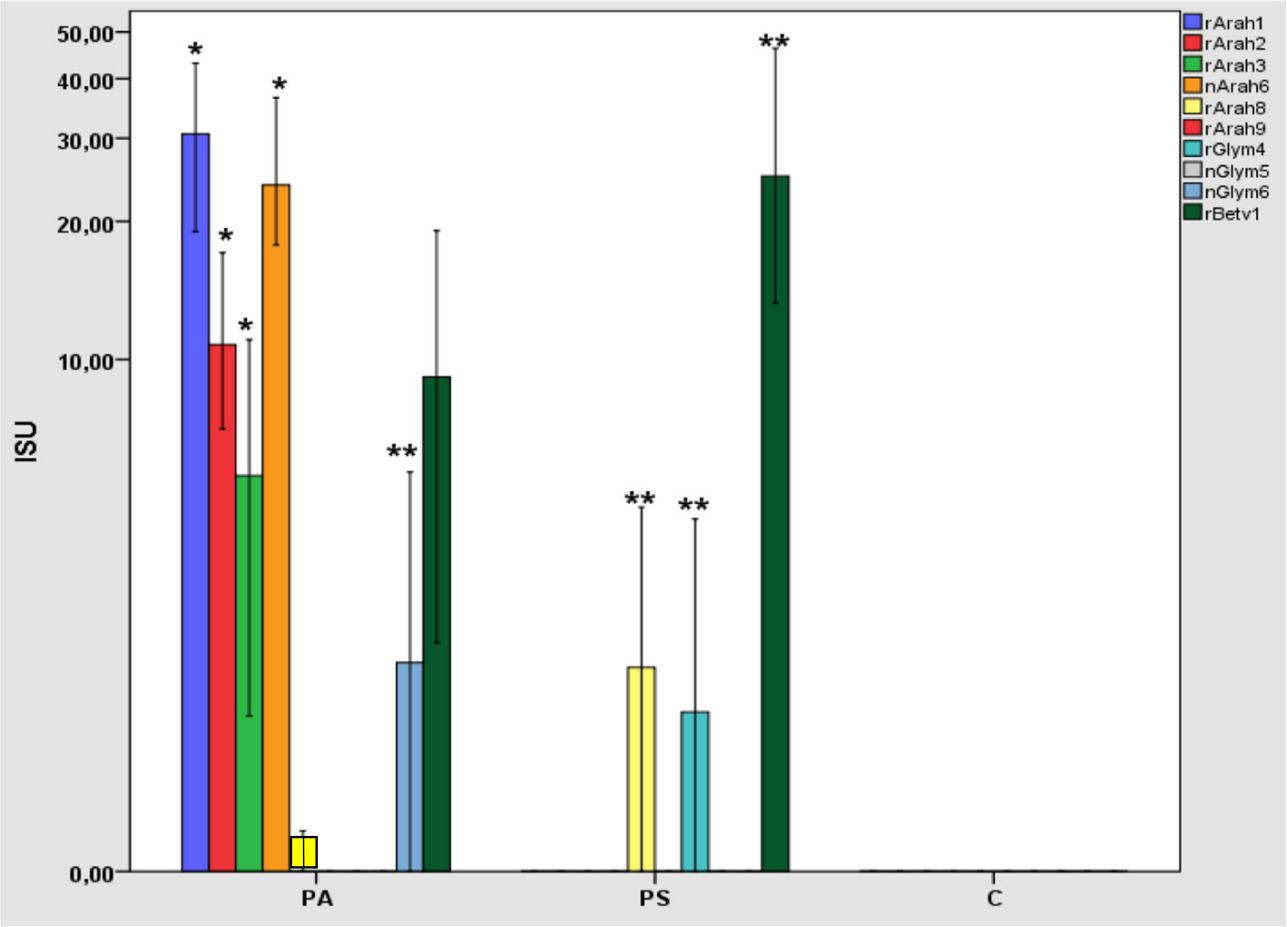
The median of specific IgE against the allergen components rAra h 1, rAra h 2, rAra h 3, nAra h 6, rAra h 8, rAra h 9, rGly m 4, nGly m 5, nGly m 6 and r Bet v 1 in patients with severe peanut allergy compared to sensitized patients and controls. PA, patients with severe peanut allergy; PS, peanut-sensitized patients; C, healthy controls; ISU, ISAC Standard Units. * *p* < 0.05, ** *p* < 0.01. The Mann-Whitney *U*-test was used for statistical comparisons of the different study groups

The median ISU values for the peanut components rAra h 1 (30.6; IQR 46.5), rAra h 2 (10.8; IQR 23.2), rAra h 3 (5.4; IQR 18.5), and nAra h 6 (23.6; IQR 41.1) in the PA-group were significantly higher (*p* < 0.001 for each component) than the corresponding values in the PS-group (Fig. 6). The median ISU for the soy component nGly m 6 1.6 ISU (IQR 8.5) was also significantly higher in the PA-group (*p* = 0.03). However, in the PS-group, the median ISU values for rAra h 8 (1.6; IQR 4,8), rBet v 1 (25; IQR 34.5), and rGly m 4 (1.2; IQR 5.2) were significantly higher (*p* = 0.01, *p* = 0.023 and *p* = 0.02, respectively), as compared to the PA-group (Fig. 6). In summary, the IgE reactivities for peanut and soy, indicative of severe allergy, were found to be significantly higher in the PA-group, whereas the levels of IgE antibodies for peanut and soy, indicative of cross-reactivity with birch pollen, were significantly higher in the PS-group.

### Frequency of IgE sensitization to allergen components

In the PA-group, all the patients were sensitized to nAra h 6 and four of these patients were mono-sensitized to nAra h 6, while one patient was sensitized to both nAra h 6 and rAra h 8. All the remaining patients in the PA-group were co-sensitized to rAra h 2 and at least one of the other allergen components of peanut (rAra h 1, 3, 8). In the PS-group, 15 patients were sensitized to Ara h 8, one patient sensitized to both rAra h 3 and rAra h 8, one patient was mono-sensitized to rAra h 9 and one patient was sensitized only to nAra h 6 and rAra h 9. Six patients were not sensitized to any of the peanut components tested but tested positive for peanut in the SPT and/or IgE assay (Table 2).

**Table 2:** IgE sensitization frequency to allergen components (>0.35 ISU) between patients allergic to peanuts and patients sensitized to peanuts. (*PA* peanut allergic patients, *PS* peanut sensitized patients)

Peanut allergens components	PA (*n* = 47)	PS (*n* = 22)
rAra h 1	40	0
rAra h 2	43	0
rAra h 3	32	1
nAra h 6	47	1
rAra h 8	15	15
rAra h 9	0	2

## Discussion

In the present study, we show that BAT is useful as a complementary tool for the diagnosis and evaluation of severe peanut allergy in adults. The study clearly shows that basophil reactivity is significantly higher in patients with a history of severe allergy to peanuts (PA), as compared with peanut-sensitized (PS) patients; with a ROC area under the curve of 0.862 and at a BAT AC50 value of 5.27 the BAT shows a specificity of 86 % and a sensitivity of 79 %. The BAT AC50 value of 5.27 corresponds to a concentration of peanut antigen of 1.8 ng/ml being used to stimulate the basophils. Interestingly, the BAT AC50 value for the PA-group only weakly correlated with the ISAC value for the peanut components rAra h 1, rAra h 2, rAra h 3 and rAra h 6, which indicates that these two tests complement each other. This suggests that BAT can serve as a complementary diagnostic tool to the conventional investigations with SPT and specific IgE for patients with suspected severe peanut allergy.

Recently, a study conducted by Santos *et al.* [24], in which children with a history of anaphylaxis to peanut were compared with peanut-sensitized children, showed that the BAT could distinguish children with severe peanut allergy from children sensitized to peanuts with a sensitivity of 97.6 % and a specificity of 96 % [24]. In that study, the majority of the subjects underwent an open challenge with peanut in addition to the BAT and conventional allergy tests. These results are in the line with the results of the present study. Other research groups have also proposed the BAT as a diagnostic tool for peanut allergy. Homsak *et al.* [28] reported that BAT reactivity values were higher in children who experienced severe reactions than in children with milder reactions. Glaumann *et al.* [25, 29] showed that children who reacted to peanuts in a DBPCFC had a higher BAT reactivity than non-reactors. In the present study, none of the patients with a history of anaphylaxis or very high IgE titers was investigated with an open challenge, so the previous results could not be confirmed [15, 16].

In recent years, the diagnostic options have expanded with the use of allergen components. In all, six allergen components for peanut allergy have been studied (rAra h 1, rAra h 2, rAra h 3, nAra h 6, rAra h 8 and rAra h 9) as a complementary diagnostic tool. However, limited information is available regarding rAra h 9, which is the predominant peanut allergen in Mediterranean regions [30]. The importance of rAra h components for predicting true peanut allergy is well documented in studies conducted on children [31–34]. Accordingly, in the present study, the patients who were diagnosed with severe peanut allergy (PA) also showed significantly higher levels of IgE to the peanut allergen components rAra h 1, rAra h 2, rAra h 3 and nAra h 6, as compared with the peanut-sensitized patients (PS). However, recently it was shown that, IgE to rAra h 2 was the best predictor of clinical peanut allergy but rAra h 2 reactivity alone could neither discriminate between mild or severe peanut allergy nor could its absence exclude peanut allergy in an adult population [35]. It has been suggested that in children, measurements of rAra h2 and nAra h 6 (as homologs) should be adequate as complementary tests [34], while in the study of Bindeslev-Jensen *et al.* [36], which was carried out in a mixed population of children and adults, it was found that IgE reactivity to rAra h 2 yielded a cut-off of 1.63 kU/L with a specificity of 100 % and sensitivity of 70 %. IgE reactivities to these peanut components have been proposed as a complementary test to provide support for a diagnosis of suspected severe peanut allergy [31–34]. However, when analyzing the reactivity to birch we clearly see that peanut-sensitized, non-anaphylactic patients (PS) show a significantly higher level of specific IgE to birch, rBet v 1 and rAra h 8, as compared with patients with severe peanut allergy, which indicates that they are sensitized to peanut due to a cross-reaction between birch pollen and peanuts [8].

In the multivariate factor analysis (SIMCA), we show that SPT peanut, IgE to peanut, peanut components (rAra h 1, rAra h 2, rAra h 3, nAra h 6), and the BAT for peanut are all predictors of severe peanut allergy. This is in contrast to the situation for the peanut-sensitized patients, where the relevant parameters are IgE to birch, rAra h 8 and rGly m 4, implying that these patients suffer from birch-related, cross-reacting mild or negligible symptoms.

It has been proposed that individuals with severe allergy to peanut may develop a clinical sensitization to legumes and *vice versa,* however there is little evidence to support the notion that patients who are allergic to peanut develop an allergy to soy because of the cross-reactivity between the proteins in the two allergens [11, 12]. On the other hand, patients who are allergic to birch pollen may also develop a clinically low-grade reactivity to soy protein due to cross-reactivity that exists between rGly m 4 in soy and the major birch pollen protein rBet v 1 [37–39]. For patients with severe reactions to soy, it has been suggested that they are sensitized to the proteins nGly m 5 and nGly m 6 [40]. This is supported by the results obtained in the present study, in that we observed that IgE directed against both nGly m 5 and nGly m6 correlated with the BAT AC50 for soy, specific IgE to soy extract and SPT to soy, which taken together suggest a clinical allergy to soy in the PA-group. It is interesting to note that the reactivities to nGly m 5 and nGly m 6 also correlated with the levels of specific IgE to peanut and peanut recombinant allergens rAra h 1, rAra h 2, rAra h 3 and nAra h 6 in the PA-group, which suggests that sensitization to soy exerts an important clinical impact. This may explain why patients with peanut allergy also show adverse reactions to soy. In contrast, in the PS-group, rGly m 4 correlated with the BAT AC50 for birch, specific IgE for birch, rBet v 1 and rAra h 8, which can be attributed to the co-existent allergy to birch pollen. rGly m 4 did not correlate with the peanut components rAra h 1, rAra h 2, rAra h 3 and nAra h 6 or with specific IgE to peanut, which implies that reactivity to this component is not related to true peanut allergy. In the PS-group, it is clear that rGly m 4 is correlated with specific IgE to birch, rAra h 8 and rBet v 1, which confirms the data concerning cross-reactivity between birch pollen and this soy protein [39]. In addition, our study clearly shows higher BAT AC50 values for soy in the PA-group than in the PS-group, which supports the idea that patients with severe allergy to peanut also have developed a more severe allergy to soy.

Analysis of combinations of the BAT AC50P, IgE to peanut, rAra h 1, rAra h 2, rAra h 3 and nAra h 6 by applying logistic a regression analysis model revealed that any combination of the different diagnostic tests does not improve the accuracy of diagnosing severe peanut allergy (data not shown). The use of the BAT before a food challenge has already been suggested by Rubio *et al.*, who observed a correlation between basophil activation and the outcome of oral food challenge before the re-introduction of cow milk in allergic children [41]. It is worth mentioning that in one patient from the PA-group who had high IgE titers to peanut (92 kU/L) and near-maximal activation of basophils to peanut (ten serial 10-fold dilutions), there were maximal basophil responses to soy and birch (ten serial 10-fold dilutions). Interestingly, this patient had low IgE titers for soy (0.97 kU/L) and birch (0.55 kU/L), which implies that the BAT may be a sensitive method for detecting potential anaphylactic responses to allergens not identified by IgE reactivity. This type of reactivity may explain some of the fatal reactions to soy observed in peanut-allergic patients not diagnosed with soy allergy [42]. In this context, it should be added that the BAT also seems to be highly sensitive and specific for detecting traces of functionally active peanut allergen even after the processing of peanut-containing foodstuffs [43].

A relatively large proportion of the healthy controls showed a low level of basophil activation to the positive control, as compared with the allergic patients. This may be explained by the fact that healthy controls have very low total IgE levels, so their basophils are less sensitive to stimulation with a cross-linking antibody to IgE and fMLP. This observation is in line with previous data obtained using the BAT for the diagnosis of food allergy in both allergic patients and healthy controls where not all of the subjects showed a clear basophil response when activated with cross-linking antibodies to IgE [18]. Similar results were obtained more recently in a study in which the BAT was evaluated in atopic and non-atopic subjects [44].

It is worth noting that the BAT is not influenced by antihistamine medication [45], and there is currently no evidence that ongoing treatment with inhaled steroids influences the BAT outcome.

The high levels of sensitivity and specificity of the BAT in identifying individuals with clinically important IgE-mediated food allergy were confirmed in a previous study in which patients allergic to birch with oral allergy syndrome (OAS) to apple were compared with birch-allergic patients without OAS to apple [46].

In the present study, it is shown that the BAT AC50 for peanut does not correlate with the levels of IgE to the peanut allergen components, which suggests that the BAT can identify patients who are allergic to peanuts and who are not diagnosed with the conventional IgE-tests. A combination of SPT, specific IgE, recombinant allergens of peanuts and the BAT may be optimal for securing an accurate diagnosis, as supported by a recent report from Spain [47].

One limitation of the present study is that patients with suspected severe allergy to peanut could not be investigated with an open challenge for ethical reasons. Therefore, a correlation between the BAT outcome and the present clinical anaphylactic status of patients is not available.

As there are still very few studies investigating the BAT as a diagnostic tool in adults with allergy to peanuts, more studies are needed to establish its diagnostic potential to predict severe reactions to peanuts.

## Conclusions

BAT may be used as a complementary diagnostic tool to ensure accurate diagnosis of severe peanut allergy in adults. Further studies to correlate BAT reactivity with the outcome of clinical challenge would be appropriate to validate the BAT test as a tool to reduce the need for open challenge in peanut allergic adults. Furthermore, the BAT may be useful in revealing a hidden yet serious allergy to soy in patients with peanut allergy.

## Additional files

Additional file 1: Table S3. Correlations between the most influential variables associated with severe peanut allergy, as revealed by the OPLS-DA analysis shown in Fig. 1b and the BAT results for peanut soy and birch. Additional file 2: Table S4. Correlations between the most influential variables associated with peanut sensitization (PS), as revealed by the OPLS-DA analysis shown in Fig. 1b and the BAT results for peanut soy and birch. Additional file 3: Figure S1. Plot of variable influence on projection (VIP) values. The VIP-values are depicted as a column plot sorted in descending order with confidence intervals derived from jack knifing. The plot indicates the relative levels of importance of the X-variables that contribute most (positively or negatively) to the association with patients with severe peanut allergy or patients who are sensitized to peanuts. The X-variables included in the final OPLS-DA plot in Fig. 3 are marked with bold text in the VIP plot. Additional file 4: Table S2. The median (range) total IgE and specific IgE expressed in kU/L. The median (range) for rAra h 1,2,3,6,8,9 which are the recombinant components for peanut, rBet v 1 for birch pollen and r Gly m 4, nGly m 5, rGly m 6 for soy are expressed in ISU (ISAC Standard Units). (PA = patients with severe allergy to peanuts, PS = peanut sensitized patients, C = healthy controls). Additional file 5: Table S1. Skin prick test response to peanut, birch and soy. Frequency of patients with a wheal diameter expressed as 1+ to 6+ (as described in the methods section) within each patient group. (PA = patients with severe allergy to peanuts, PS = peanut sensitized patients).

## Abbreviations

PA-group: Patients with severe peanut allergy
PS-group: Peanut-sensitized patients
C-group: Healthy control subjects
BAT: Basophil activation test
OAS: Oral allergy syndrome
fMLP: N-formyl-Met-Leu-Phe
BAT AC50: Basophil allergen threshold sensitivity, as the lowest concentration of allergen was able to activate 50 % of the basophils that were activated in the stimulation control for each patient
