# Supplementary material for: Use of a basophil activation test as a complementary diagnostic tool in the diagnosis of severe peanut allergy in adults
Source: Clin Transl Allergy. 2015 Jun 11;5:22. doi: 10.1186/s13601-015-0064-9 (PMC4464723; doi:10.1186/s13601-015-0064-9)
Supplement: Additional file 1: Table S3. — Correlations between the most influential variables associated with severe peanut allergy, as revealed by the OPLS-DA analysis shown in Fig. 1b and the BAT results for peanut soy and birch. [file 13601_2015_64_MOESM1_ESM.pdf]

Table 3S

Correlations in the PA-group with the Spearman's Correlation Test

			SPT Peanut	IgE Peanut	IgE Soy	BAT AC50 Peanut	BAT AC50 Soy	BAT AC50 Birch	rAra h 1	rAra h 2	rAra h 3	nAra h 6	nGly m 5	nGly m 6
Spearman's rho	**SPT Peanut**	Correlation Coefficient	1,000	,061	-,057	-,022	-,041	-,100	-,027	,070	,000	,121	-,140	-,114
		Sig. (2-tailed)	.	,682	,705	,882	,787	,505	,855	,639	,999	,420	,349	,447
		Number of patients	47	47	47	47	47	47	47	47	47	47	47	47
	**IgE Peanut**	Correlation Coefficient	,061	1,000	,770**	,235	,584**	-,044	,846**	,752**	,809**	,784**	,558**	,652**
		Sig. (2-tailed)	,682	.	,000	,112	,000	,769	,000	,000	,000	,000	,000	,000
		Number of patients	47	47	47	47	47	47	47	47	47	47	47	47
	**IgE Soy**	Correlation Coefficient	-,057	,770**	1,000	,099	,585**	,184	,677**	,612**	,823**	,609**	,659**	,923**
		Sig. (2-tailed)	,705	,000	.	,507	,000	,215	,000	,000	,000	,000	,000	,000
		Number of patients	47	47	47	47	47	47	47	47	47	47	47	47
	**BAT AC50 Peanut**	Correlation Coefficient	-,022	,235	,099	1,000	,413**	,161	,314*	,291*	,289*	,347*	,036	,068
		Sig. (2-tailed)	,882	,112	,507	.	,004	,280	,032	,047	,049	,017	,813	,652
		Number of patients	47	47	47	47	47	47	47	47	47	47	47	47
	**BAT AC50 Soy**	Correlation Coefficient	-,041	,584**	,585**	,413**	1,000	,268	,508**	,476**	,661**	,484**	,391**	,583**
		Sig. (2-tailed)	,787	,000	,000	,004	.	,068	,000	,001	,000	,001	,007	,000
		Number of patients	47	47	47	47	47	47	47	47	47	47	47	47
	**BAT AC50 Birch**	Correlation Coefficient	-,100	-,044	,184	,161	,268	1,000	,086	,107	,130	,108	,221	,304*
		Sig. (2-tailed)	,505	,769	,215	,280	,068	.	,565	,476	,384	,469	,135	,037
		Number of patients	47	47	47	47	47	47	47	47	47	47	47	47
	**rAra h 1**	Correlation Coefficient	-,027	,846**	,677**	,314*	,508**	,086	1,000	,777**	,796**	,804**	,459**	,603**
		Sig. (2-tailed)	,855	,000	,000	,032	,000	,565	.	,000	,000	,000	,001	,000
		Number of patients	47	47	47	47	47	47	47	47	47	47	47	47
	**rAra h 2**	Correlation Coefficient	,070	,752**	,612**	,291*	,476**	,107	,777**	1,000	,742**	,929**	,367*	,543**
		Sig. (2-tailed)	,639	,000	,000	,047	,001	,476	,000	.	,000	,000	,011	,000
		Number of patients	47	47	47	47	47	47	47	47	47	47	47	47
	**rAra h 3**	Correlation Coefficient	,000	,809**	,823**	,289*	,661**	,130	,796**	,742**	1,000	,741**	,632**	,791**
		Sig. (2-tailed)	,999	,000	,000	,049	,000	,384	,000	,000	.	,000	,000	,000
		Number of patients	47	47	47	47	47	47	47	47	47	47	47	47
	**nAra h 6**	Correlation Coefficient	,121	,784**	,609**	,347*	,484**	,108	,804**	,929**	,741**	1,000	,389**	,547**
		Sig. (2-tailed)	,420	,000	,000	,017	,001	,469	,000	,000	,000	.	,007	,000
		Number of patients	47	47	47	47	47	47	47	47	47	47	47	47
	**nGly m 5**	Correlation Coefficient	-,140	,558**	,659**	,036	,391**	,221	,459**	,367*	,632**	,389**	1,000	,701**
		Sig. (2-tailed)	,349	,000	,000	,813	,007	,135	,001	,011	,000	,007	.	,000
		Number of patients	47	47	47	47	47	47	47	47	47	47	47	47
	**nGly m 6**	Correlation Coefficient	-,114	,652**	,923**	,068	,583**	,304*	,603**	,543**	,791**	,547**	,701**	1,000
		Sig. (2-tailed)	,447	,000	,000	,652	,000	,037	,000	,000	,000	,000	,000	.
		Number of patients	47	47	47	47	47	47	47	47	47	47	47	47

**. Correlation is significant at the 0.01 level (2-tailed).

*. Correlation is significant at the 0.05 level (2-tailed).
