# Supplementary material for: Use of a basophil activation test as a complementary diagnostic tool in the diagnosis of severe peanut allergy in adults
Source: Clin Transl Allergy. 2015 Jun 11;5:22. doi: 10.1186/s13601-015-0064-9 (PMC4464723; doi:10.1186/s13601-015-0064-9)
Supplement: Additional file 2: Table S4. — Correlations between the most influential variables associated with peanut sensitization (PS), as revealed by the OPLS-DA analysis shown in Fig. 1b and the BAT results for peanut soy and birch. [file 13601_2015_64_MOESM2_ESM.pdf]

Table 4S.

Correlations in the PS-group with the Spearman's Correlation Test

			IgE Birch	BAT AC50 Peanut	BAT AC50 Soy	BAT AC50 Birch	rAra h 8	rGly m 4
Spearman's rho	**IgE Birch**	Correlation Coefficient	1,000	,418	,192	,258	,795**	,738**
		Sig. (2-tailed)	.	,053	,391	,246	,000	,000
		Number of patients	22	22	22	22	22	22
	**BAT AC50 Peanut**	Correlation Coefficient	,418	1,000	,689**	,735**	,479*	,638**
		Sig. (2-tailed)	,053	.	,000	,000	,024	,001
		Number of patients	22	22	22	22	22	22
	**BAT AC50 Soy**	Correlation Coefficient	,192	,689**	1,000	,442*	,373	,447*
		Sig. (2-tailed)	,391	,000	.	,040	,088	,037
		Number of patients	22	22	22	22	22	22
	**BAT AC50 Birch**	Correlation Coefficient	,258	,735**	,442*	1,000	,362	,372
		Sig. (2-tailed)	,246	,000	,040	.	,098	,088
		Number of patients	22	22	22	22	22	22
	**rAra h 8**	Correlation Coefficient	,795**	,479*	,373	,362	1,000	,757**
		Sig. (2-tailed)	,000	,024	,088	,098	.	,000
		Number of patients	22	22	22	22	22	22
	**rGly m 4**	Correlation Coefficient	,738**	,638**	,447*	,372	,757**	1,000
		Sig. (2-tailed)	,000	,001	,037	,088	,000	.
		Number of patients	22	22	22	22	22	22

**. Correlation is significant at the 0.01 level (2-tailed).

*. Correlation is significant at the 0.05 level (2-tailed).
