# Supplementary figures and images for: Use of a basophil activation test as a complementary diagnostic tool in the diagnosis of severe peanut allergy in adults
Source: Clin Transl Allergy. 2015 Jun 11;5:22. doi: 10.1186/s13601-015-0064-9 (PMC4464723; doi:10.1186/s13601-015-0064-9)

Figure 1S.

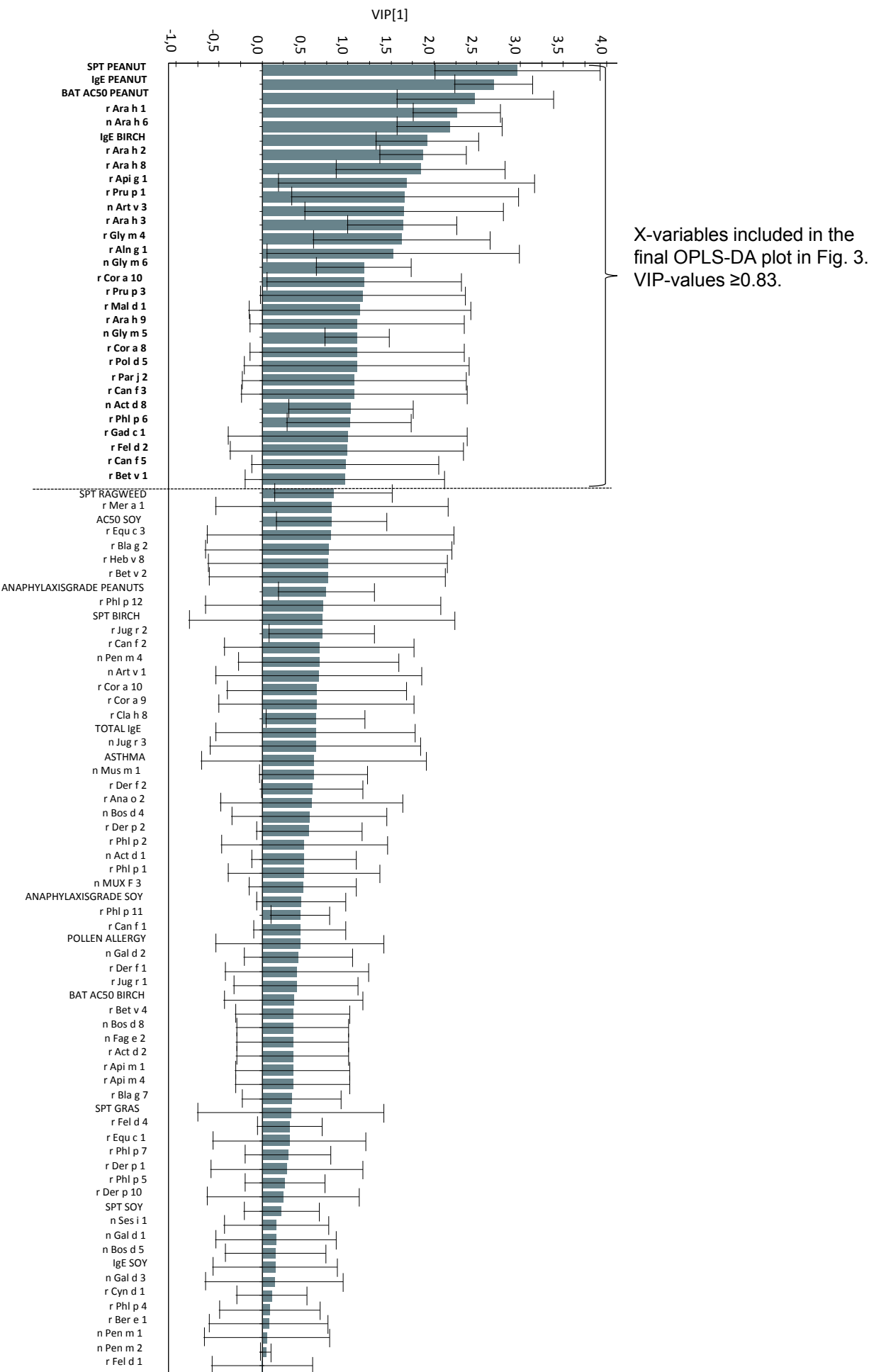

Supplement: Additional file 3: Figure S1. — Plot of variable influence on projection (VIP) values. The VIP-values are depicted as a column plot sorted in descending order with confidence intervals derived from jack knifing. The plot indicates the relative levels of importance of the X-variables that contribute most (positively or negatively) to the association with patients with severe peanut allergy or patients who are sensitized to peanuts. The X-variables included in the final OPLS-DA plot in Fig. 3 are marked with bold text in the VIP plot. [file 13601_2015_64_MOESM3_ESM.pdf]
