# Supplementary material for: Use of a basophil activation test as a complementary diagnostic tool in the diagnosis of severe peanut allergy in adults
Source: Clin Transl Allergy. 2015 Jun 11;5:22. doi: 10.1186/s13601-015-0064-9 (PMC4464723; doi:10.1186/s13601-015-0064-9)
Supplement: Additional file 4: Table S2. — The median (range) total IgE and specific IgE expressed in kU/L. The median (range) for rAra h 1,2,3,6,8,9 which are the recombinant components for peanut, rBet v 1 for birch pollen and r Gly m 4, nGly m 5, rGly m 6 for soy are expressed in ISU (ISAC Standard Units). (PA = patients with severe allergy to peanuts, PS = peanut sensitized patients, C = healthy controls). [file 13601_2015_64_MOESM4_ESM.docx]

**Table 2S**

| **IgE-reactivity (median)** | **PA (n=47)** | **PS (n=22)** | **C (n=22)** |
| --- | --- | --- | --- |
| **Total IgE** | 380.0 (3-1800) | 200.0 (13-4600) | 17.5 (2-140) |
| **IgE peanut** | 64 (0.55-100) | 2.45 kU/L (0.1-15) | <0.35 (0.0-0.05) |
| **IgE birch** | 6.1 (0.0-100) | 25.0 kU/L (0.0-100) | <0.35 (0.0-0.24) |
| **IgE soy** | 1.4 (0.0-40) | 0.36 kU/L (0-40) | <0.35 (0.0-0.14) |
| **rAra h 1** | 30.55 (0.0-138.71) | <0.3 | <0.3 |
| **rAra h 2** | 10.75 (0.0-1117.58) | <0.3 | <0.3 |
| **rAra h 3** | 5.38 (0.0-64.40) | <0.3 | <0.3 |
| **nAra h 6** | 23.55 (1.21-178.24) | <0.3 | <0.3 |
| **rAra h 8** | <0.3 | 1.60 (0.0-17.80) | <0.3 |
| **rAra h 9** | <0.3 | <0.3 | <0.3 |
| **rGly m 4** | <0.3 | 1.11 (0.0-27.88) | <0.3 |
| **nGly m 5** | <0.3 | <0.3 | <0.3 |
| **nGly m 6** | 1.62 (0.0-53.60) | <0.3 | <0.3 |
| **rBet v 1** | 8.85 (0.0-100) | 24.96 (0.0-168.42) | <0.3 |

**Table 2S.** The median (range) total IgE and specific IgE expressed in kU/L. The median (range) for rAra h 1,2,3,6,8,9 which are the recombinant components for peanut, rBet v 1 for birch pollen and r Gly m 4, nGly m 5, rGly m 6 for soy are expressed in ISU (ISAC Standard Units). (PA= patients with severe allergy to peanuts, PS=peanut sensitized patients, C=healthy controls)
