# Supplementary material for: Use of a basophil activation test as a complementary diagnostic tool in the diagnosis of severe peanut allergy in adults
Source: Clin Transl Allergy. 2015 Jun 11;5:22. doi: 10.1186/s13601-015-0064-9 (PMC4464723; doi:10.1186/s13601-015-0064-9)
Supplement: Additional file 5: Table S1. — Skin prick test response to peanut, birch and soy. Frequency of patients with a wheal diameter expressed as 1+ to 6+ (as described in the methods section) within each patient group. (PA = patients with severe allergy to peanuts, PS = peanut sensitized patients). [file 13601_2015_64_MOESM5_ESM.doc]

**Table 1S**

| **SPT (peanut** | **PA (n=47)** | **PS (n=22** |
| --- | --- | --- |
| **1+** | 1 | 2 |
| **2+** | 0 | 8 |
| **3+** | 7 | 4 |
| **4+** | 17 | 4 |
| **5+** | 14 | **-** |
| **6+** | 8 | **-** |
| **SPT (birch)** |  |  |
| **1+** | 6 | 3 |
| **2+** | 5 | 2 |
| **3+** | 19 | 7 |
| **4+** | 3 | 4 |
| **5+** | 1 | 2 |
| **6+** | **-** | **-** |
| **SPT (soy)** |  |  |
| **1+** | 6 | 9 |
| **2+** | 7 | 9 |
| **3+** | 5 | 2 |
| **4+** | **-** | **-** |
| **5+** | **-** | **-** |
| **6+** | **-** | **-** |

**Table 1S**. Skin prick test response to peanut, birch and soy. Frequency of patients with a wheal diameter expressed as 1+ to 6+ (as described in the methods section) within each patient group. (PA=patients with severe allergy to peanuts, PS=peanut sensitized patients)
